# High fat diet-induced obesity prolongs critical stages of the spermatogenic cycle in a Ldlr^−/−^.Leiden mouse model

**DOI:** 10.1038/s41598-021-04069-y

**Published:** 2022-01-11

**Authors:** D. Komninos, L. Ramos, G. W. van der Heijden, M. C. Morrison, R. Kleemann, A. E. van Herwaarden, A. J. Kiliaan, I. A. C. Arnoldussen

**Affiliations:** 1 Department of Obstetrics and Gynaecology, Radboud University Medical Center, Geert Grooteplein Zuid 10, 6525 GA Nijmegen, The Netherlands; 2 Department of Metabolic Health Research, Netherlands Organisation for Applied Scientific Research (TNO), Zernikedreef 9, 2333 CK Leiden, The Netherlands; 3 Department of Human and Animal Physiology, Wageningen University, De Elst 1, 6708 WD Wageningen, The Netherlands; 4 Department of Laboratory Medicine, Radboud University Medical Center, Geert Grooteplein Zuid 10, 6525 GA Nijmegen, The Netherlands; 5 Department of Medical Imaging, Anatomy, Donders Institute for Brain, Cognition and Behaviour, Preclinical Imaging Center PRIME, Radboud University Medical Center, Geert Grooteplein Noord 21, 6525 EZ Nijmegen, The Netherlands

**Keywords:** Metabolic disorders, Structural biology

## Abstract

Obesity can disturb spermatogenesis and subsequently affect male fertility and reproduction. In our study, we aim to elucidate at which cellular level of adult spermatogenesis the detrimental effects of obesity manifest. We induced high fat diet (HFD) obesity in low-density lipoprotein receptor knock-out Leiden (Ldlr^−/−^.Leiden) mice, and studied the morphological structure of the testes and histologically examined the proportion of Sertoli cells, spermatocytes and spermatids in the seminiferous tubules. We examined sperm DNA damage and chromatin condensation and measured plasma levels of leptin, testosterone, cholesterol and triglycerides. HFD-induced obesity caused high plasma leptin and abnormal testosterone levels and induced an aberrant intra-tubular organisation (ITO) which is associated with an altered spermatids/spermatocytes ratio (2:1 instead of 3:1). Mice fed a HFD had a higher level of tubules in stages VII + VIII in the spermatogenic cycle. The stages VII + VII indicate crucial processes in spermatogenic development like initiation of meiosis, initiation of spermatid elongation, and release of fully matured spermatids. In conclusion, HFD-induced obese Ldlr^−/−^.Leiden mice develop an aberrant ITO and alterations in the spermatogenic cycle in crucial stages (stages VII and VII). Thereby, our findings stress the importance of lifestyle guidelines in infertility treatments.

## Introduction

The impact of obesity on female fertility has been described thoroughly, and obesity has been associated with menstrual disorders, anovulation, polycystic ovarian syndrome, increased risk of miscarriage and reduced conception rate^1–3^. In slight contrast, the research field addressing the impact of obesity in male fertility is under-explored, whereas male fertility is also compromised in obese men^4–6^. Specifically male obesity is associated with decreased sperm quantity and quality, consequently resulting in decreased pregnancy rates and increased pregnancy loss in assisted reproduction^4,6,7^. Obesity can affect spermatogenesis via several mechanisms and processes, among others via hormonal changes. For instance leptin, a hormone produced by adipose tissue, inhibits the androgen production of Leydig cells in the testis^8–10^. Thereby, long-term exposure to high leptin levels can eventually lead to a decreased testosterone production^8,9^. Secondly, oestrogen levels are elevated in men with obesity, which is associated with a reduced testis size and low sperm quantity and quality^8,11^. Specifically, aromatase in adipose tissue converts testosterone into oestradiol, which results in elevated oestrogen levels in obese men^4,8,12,13^. Moreover, oestrogen inhibits the testosterone production via the hypothalamic–pituitary–gonadal (HPG) axis^9^. Therewith, excessive amounts of adipose tissue can decrease testosterone concentrations and thereby spermatogenesis through increase of leptin and oestrogen. Thirdly, in men with obesity the testicular temperature may be increased due to excessive adipose tissue stored in the testes^4,13–16^. This may result in increased apoptosis and DNA damage in developing sperm cells due to excessive levels of oxidative stress and/or an abnormal chromatin condensation^17–21^. In line with this hypothesis, some studies have indeed shown that obesity leads to higher proportion of sperm with DNA fragmentation^22–24^. However, research examining male fertility in obesity is rather limited compared to studies in women and results are rather ambiguous. In more detail, previous studies have demonstrated the adverse effects of obesity on one or more semen parameters, like ejaculate volume, sperm concentration, sperm motility and morphology^4,7,22–28^, whereas some other authors reported no significant alterations in spermatogenesis of individuals with obesity^14,29–32^. Testicular biopsies are necessary to examine cellular alterations in spermatogenesis changes in men, which is a limitation in this type of research. We examined the effect of high fat diet (HFD)-induced obesity in low-density lipoprotein receptor knock-out Leiden (LDLr^−/−^.Leiden) mice. In particular, we aimed to clarify the HFD-induced cellular alterations in adult spermatogenesis, and assessed at which level of spermatogenesis the detrimental effects of high fat diet (HFD)-induced obesity manifest^33,34^. This study may provide insight into the biological understanding of decreased male fertility in obesity which stresses the importance of lifestyle guidelines in male infertility treatment.

## Results

### HFD-fed mice had a significant higher body weight, body fat and plasma leptin levels

After 24-weeks of HFD-feeding, both body weight and body fat were significantly increased in HFD-fed Ldlr^−/−^.Leiden mice compared to mice on control diet. In more detail, the HFD-fed mice were 31.04% heavier and had 31.66% more body fat than mice in the control group (Table 1). Accordingly, plasma leptin levels of HFD fed mice were significantly higher (2.4 times) than mice on control diet (Table 1). HFD fed mice showed higher mean level of total testosterone compared to control group and overall testosterone levels varied a lot between the mice, independent of dietary group. No significant difference between dietary groups in testosterone levels (Table 1).

**Table 1:** Metabolic parameters.

Parameter (36 weeks of age)	Control	HFD
Body weight (g)	37.82 ± 1.23	49.56 ± 0.90***
Body fat (%)	8.78 ± 0.48	11.56 ± 0.58***
Plasma cholesterol (mM)	8.65 ± 0.38	28.17 ± 2.41***
Plasma triglycerides (mM)	1.20 ± 0.06	2.87 ± 0.44***
Plasma leptin (ng/mL)	21.46 ± 3.04	50.73 ± 2.60***
Plasma testosterone (nnmol/L) (median (IQR))	5.86 (7.41)	5.23 (73.69)

*p < 0.05, ***p < 0.001^35^.

*IQR* Interquartile range.

### HFD fed mice showed aberrant intra-tubular organisation

**Figure 1:**
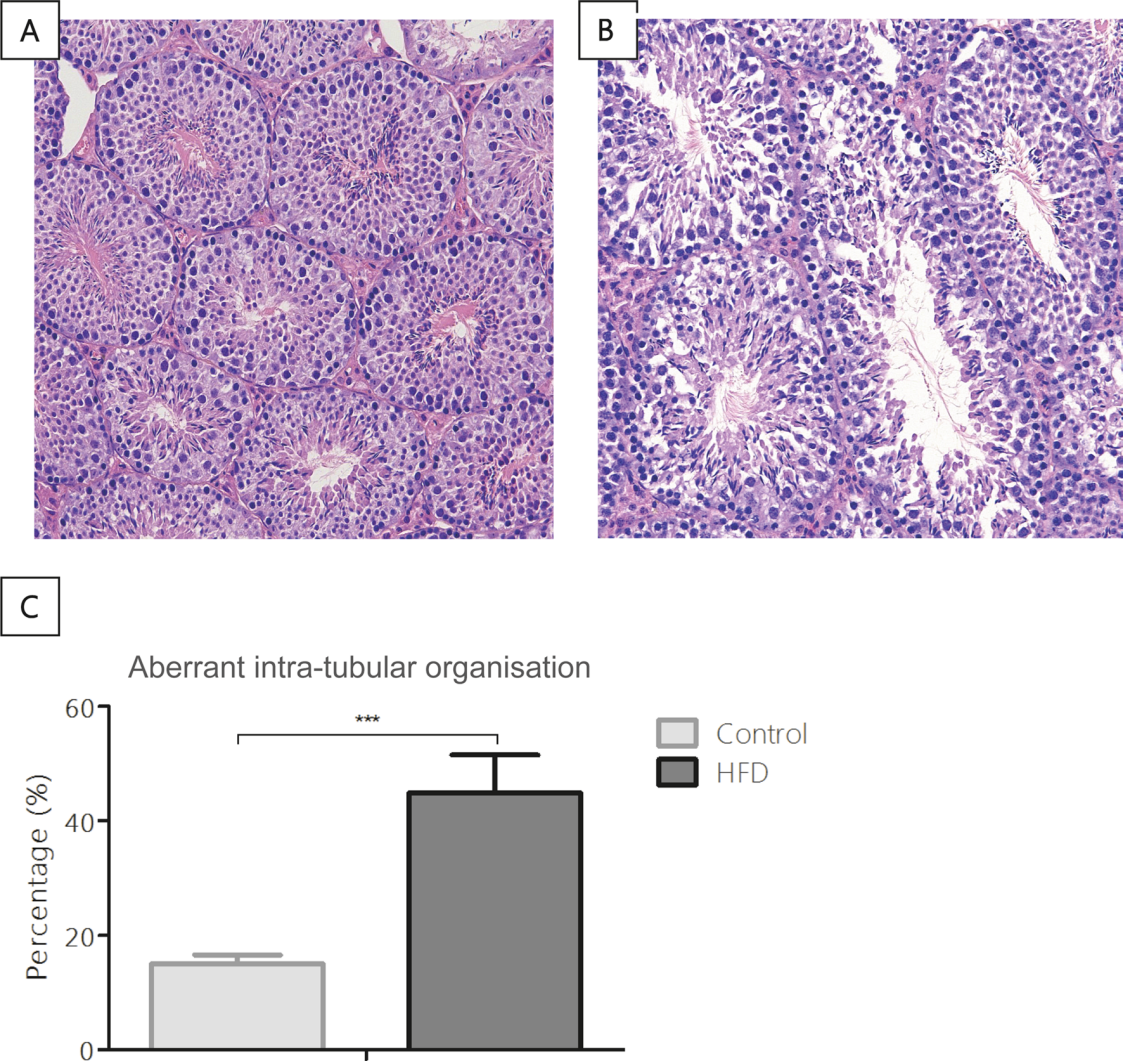
Analyses and representative images of testis morphology. (**A**) Normal tubules. (**B**) Tubules with an aberrant intra-tubular organisation. (**C**) Quantification of the seminiferous tubules (in total 52 tubules per mouse) with an aberrant intra-tubular organisation. Original magnification × 200. Control n = 13. HFD n = 14. ***p < 0.001.

We performed histological analyses of the testes to evaluate whether the HFD-induced obesity affected the organisation of the seminiferous tubules. No significant differences were found in the testis weight and volume between the two dietary groups (Supplementary Table S1). Nor did we find significant differences in the relative and absolute number of seminiferous tubules in the testis and in the thickness of the seminiferous epithelium (Supplementary Table S1). Nevertheless, the analysis of the different types of atypical tubules, a difference in aberrant intra-tubular organisation (ITO) was found. In more detail, the percentage of aberrant ITO tubules was 2.8 times higher in the testes of the HFD fed mice compared to mice fed the reference diet (p < 0.001; Fig. 1A–C). In more detail, mice in the reference group showed an average of 16% aberrant ITO tubules, whereas the HFD-fed mice had an average of 44%. Moreover, the percentage of tubules with aberrant ITO showed a strong positive correlation with the body weights of the mice (r = 0.69, p < 0.001, n = 27) and with the plasma leptin levels (r = 0.71, p < 0.001, n = 27). Interestingly, aberrant ITO tubules were negatively correlated with the spermatids/spermatocytes ratio (r = − 0.48, p = 0.04, n = 19). In addition, in normal developed tubules the spermatids/spermatocytes ratio is 3:1, while in tubules with an aberrant germ-cell organisation the ratio is 2:1. All in all, these findings indicated that HFD-induced obesity significantly increased the level of aberrant ITO of the seminiferous tubules and significantly lowered the spermatids/spermatocytes ratio.

### Number of Sertoli cells is strongly associated with number of developed spermatids

**Figure 2:**
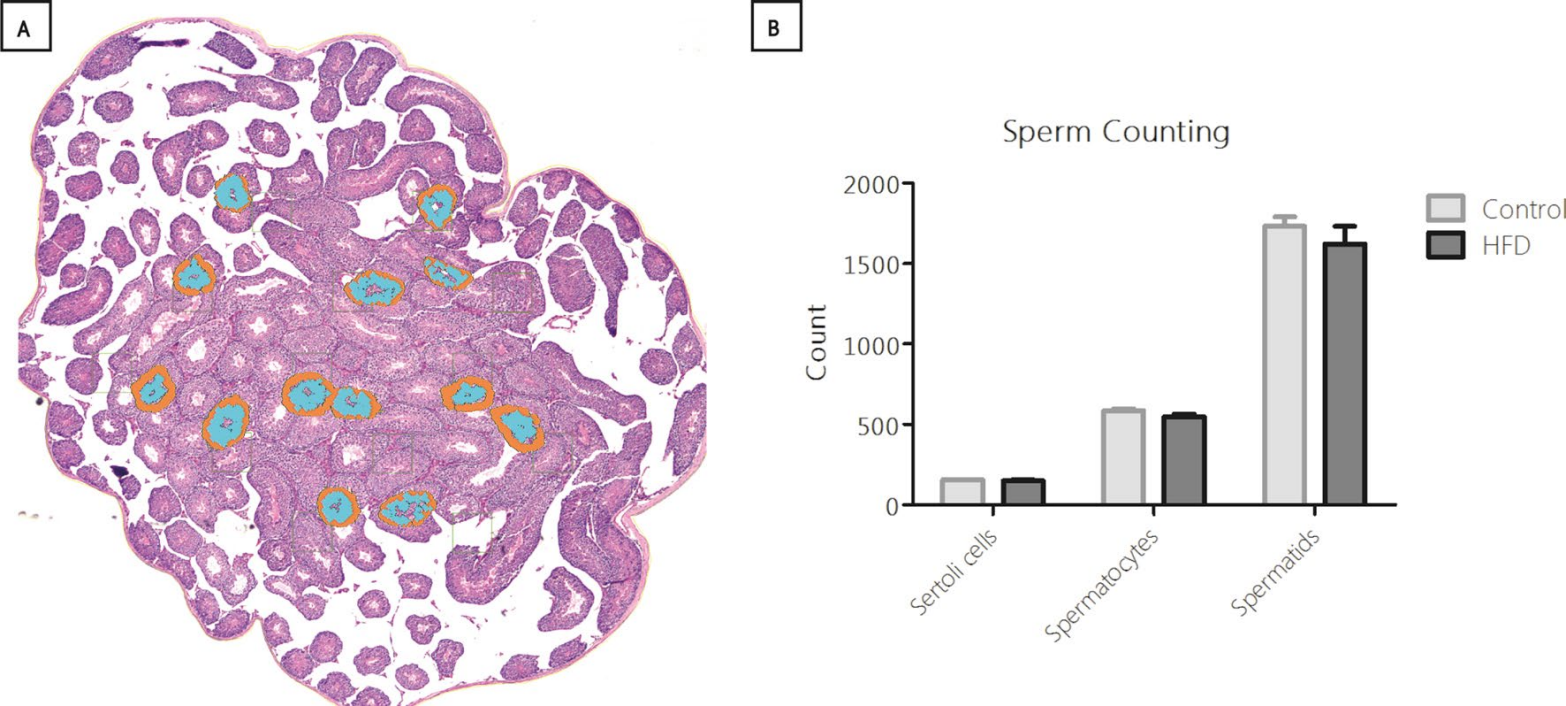
Analysis of sperm cells and Sertoli cells. (**A**) Overview of the analysis in one section, per testis four randomly selected sections were counted. Magnification × 25. (**B**) Quantification of Sertoli cells, spermatocytes, and spermatids per testis. Control n = 10. HFD n = 10.

The number of Sertoli cells, primary spermatocytes and elongating spermatids was not significantly different between dietary groups (Fig. 2). This was also the case for the ratio of elongating spermatids over spermatocytes. Nevertheless, a strong positive correlation was found between the number of Sertoli cells and spermatogonia (r = 0.74, p < 0.01, n = 19), and secondly, between Sertoli cells and the ratio of spermatids/spermatocytes (r = 0.61, p < 0.01, n = 19). Thus, we observed that the total number of Sertoli cells was strongly associated with the number of developed spermatids, which confirms the supportive function of Sertoli cells during spermatogenesis.

### HFD-feeding delayed the spermatogenic cycle in stage VII + VIII

**Figure 3:**
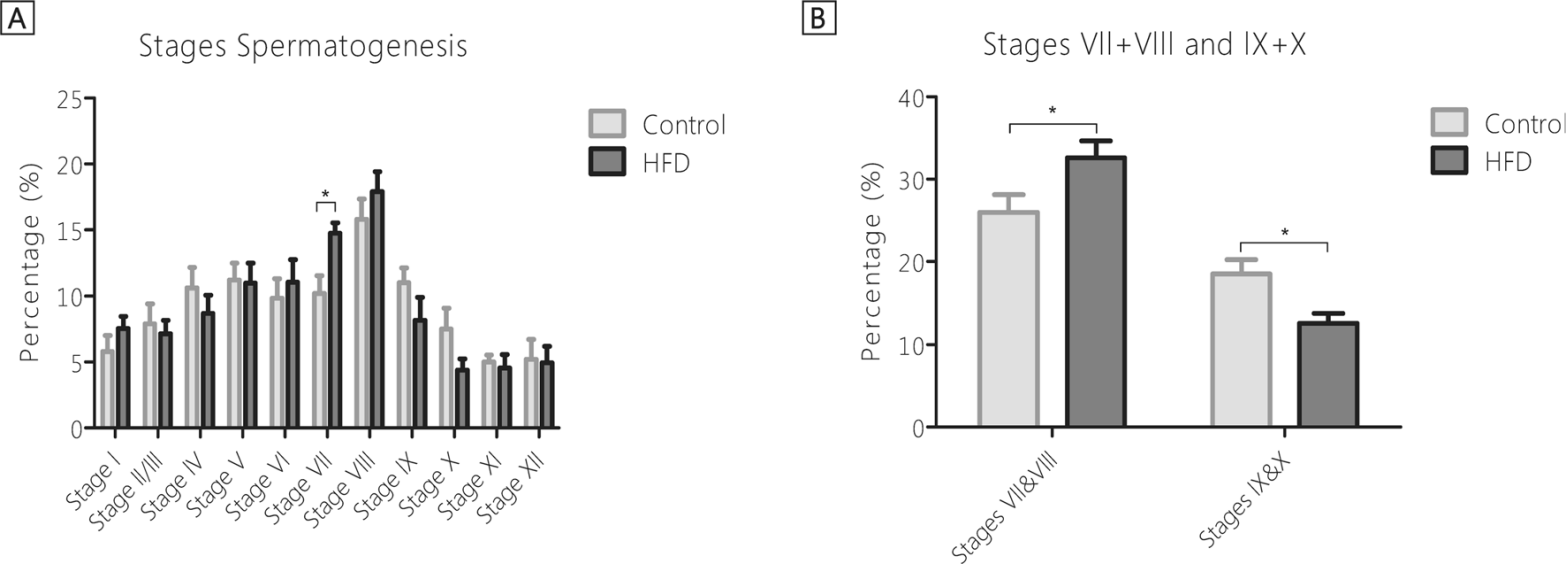
Stages of spermatogenesis in seminiferous tubules. (**A**) Analysis of the seminiferous stages, displayed as percentage of the total number of seminiferous tubules analysed. (**B**) Analysis of the stages VII & VIII and IX & X, displayed as percentage of the total number of seminiferous tubules analysed. Control n = 10. HFD n = 10. *p < 0.05.

To determine whether the HFD-induced obesity affected a specific developmental stage of spermatogenesis, we examined the developmental stages of 52 seminiferous tubules per testis per mouse. We found that the spermatogenic cycle stage per seminiferous tubule (stage I-XII) and the distribution of the seminiferous stages was significantly different between dietary groups. Specifically, HFD-fed mice had significantly more seminiferous tubules in stage VII (14.73%) than mice in the control group (10.21%, p = 0.01, Fig. 3A). As elongated spermatozoa are released during stage VIII and the difference between stage VIII and IX can be accurately determined based on the presence of spermatozoa. Thereby, we combined stages VII and VIII and stages IX and X to examine, whether the release of spermatozoa differed between dietary groups (Fig. 3B). We found that HFD fed mice had significantly more tubules in stages VII + VIII (32.6%, p = 0.041) and consequently less tubules continued to stages IX + X (12.6%, p = 0.014), when compared to mice fed the reference diet (26.0%, stage VII + VIII, and 18.5% in stage IX + X). Moreover, the combined stages VII + VIII also positively correlated with body weight (r = 0.60, p < 0.01, n = 19) and plasma leptin levels (r = 0.59, p < 0.01, n = 19). Additionally, the combined stages IX + X also showed a mild negative correlation with the percentage aberrant ITO tubules (r = − 0.52, p < 0.01, n = 19). Altogether, these findings showed that HFD-induced obesity affected the spermatogenic cycle in stage VII + VIII.

### HFD did not affect DNA damage in the testis and chromatin condensation in spermatozoa

**Figure 4:**
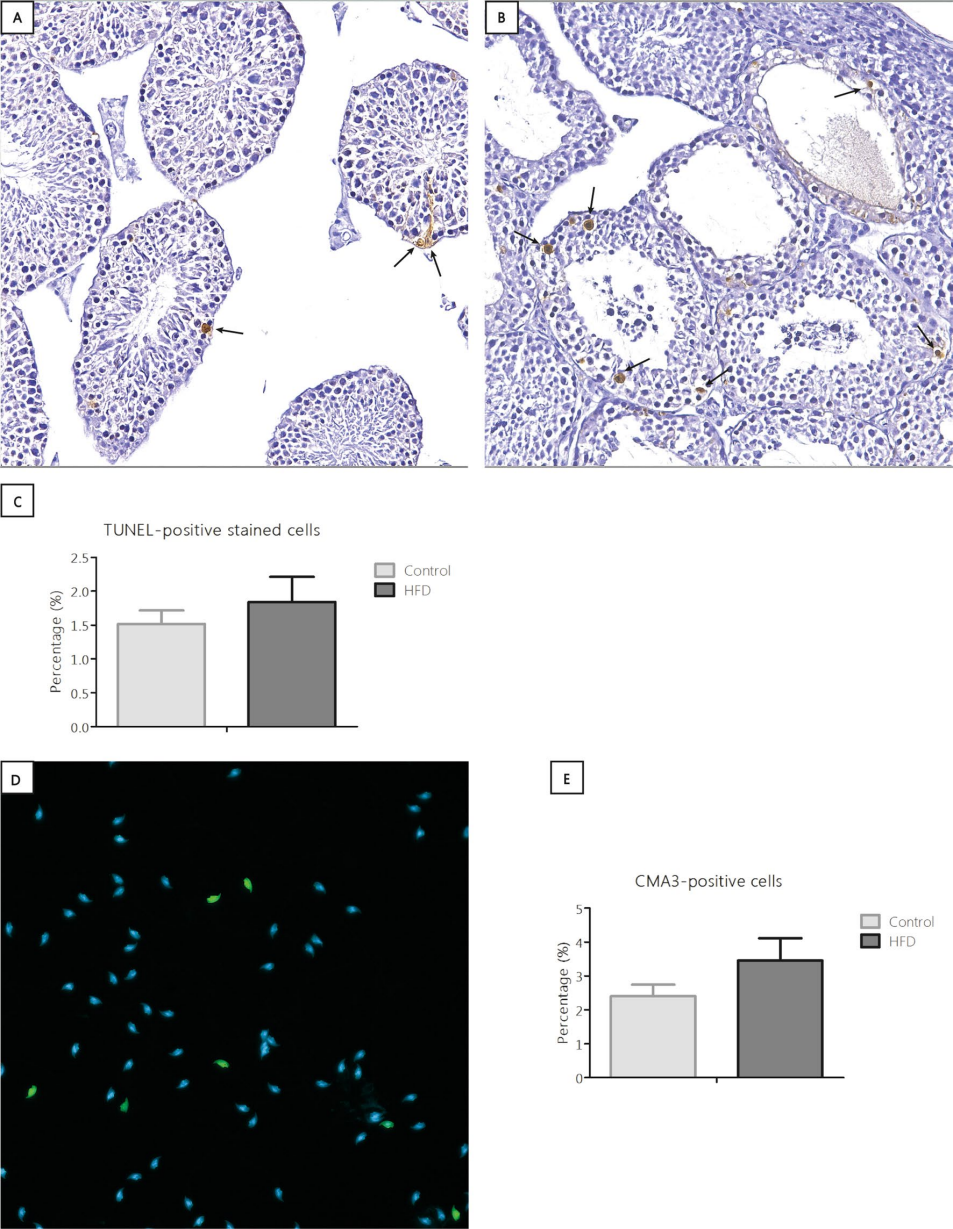
Analysis and representative images of the TUNEL and CMA3 staining. (**A**) Testis section with few TUNEL-positive cells (indicated with arrows). (**B**) Testis section with many TUNEL-positive cells. Original magnifications × 200. (**C**) Analysis of the percentage TUNEL-positive stained cells in the testes. Control n = 13. HFD n = 14. (**D**) Example of an overlay CMA3 staining with the CMA3-positive spermatozoa (green) and the CMA3-negative spermatozoa (DAPI, blue). See supplementary figure S3 for the separate channels of the overlay. (**E**) Analysis of the percentage CMA3-positive stained cells in the testes. Original magnifications × 400. HFD n = 9, Control n = 7.

No significant differences in TUNEL + levels were observed between dietary groups for either the total number or numbers per germ cell type (p = 0.44, F(1, 28) = 0.61, Fig. 4A–C), nor for the number of CMA3 + spermatozoa (p = 0.22, F(1, 13) = 1.67, Fig. 4D,E).

## Discussion

In this study, we investigated the impact of HFD-induced obesity on spermatogenesis in Ldlr^−/−^.Leiden mice. After 24 weeks of HFD feeding, these mice developed increased body weight, excessive storage of white adipose tissue, hypercholesteremia, hyperlipidaemia and high plasma leptin levels. These findings are all in line with previous research in this mouse model^35–37^. Notably, we found that HFD-induced obesity induced the significant higher formation of seminiferous tubules with aberrant intra-tubular organisation, which was associated with an altered spermatids/spermatocytes ratio (2:1 instead of 3:1). In addition, HFD feeding induced a higher level of tubules in developmental stage VII and VIII, which could indicate a prolonged spermiation in stage VII and VIII.

We found no significant difference in plasma testosterone levels in HFD-induced obese Ldlr^−/−^.Leiden mice, which is in contrast with other studies^38^. Still, some exceptionally high testosterone levels were found in specifically the HFD-fed mice, which were not related to other findings for instance aberrant intra-tubular organisation. It could be hypothesized that constant high levels of leptin starting at young age could lead to a testicular leptin resistance and thereby alter testosterone production. This has been observed in a previous study on the effects of leptin in rats^39^. These findings might be explained by a change in lipid composition due to HFD-feeding, particularly by an increase in mono-unsaturated fatty acids (MUFA)^40^. A previous study examining lipid composition in the testis and testosterone production has demonstrated that MUFA’s, contradictory to poly unsaturated fatty acids (PUFA’s), increase testosterone production^41^. More specifically, it has been shown that MUFA’s increase free testosterone levels, possibly by decreasing the binding to Sex hormone-binding globulin (SHBG)^41,42^. Thus, the testosterone production might be affected by testicular leptin resistance and/or the increase of MUFA’s. Future research in this mouse model should clarify the underlying processes and biological mechanisms involved.

To note, the testes of the HFD-induced obese mice showed significantly more aberrant ITO than the testes of the control group. Hyperleptinemia has been proven to directly impair the testicular structure^43^. The impaired testicular structure could be the result of an impaired blood-testis-barrier (BTB), which could result in decreased Sertoli cell functioning and sperm cell development^10,44^. Hyperleptinemia could disrupt cell adhesion between spermatogenic cells and Sertoli cells, which could be the origin of the observed aberrant ITO^45,46^. Moreover, hyperleptinemia may affect functioning of Sertoli cells via alterations in the HPG axis, and via lactate and acetate production^44^ causing impaired germ cell nourishing^8,47,48^. A diet abundant in unsaturated fatty acids can lower membrane fluidity, which can lead to compromised function of the cellular membrane^49,50^. The increased membrane rigidity could have contributed to the observed atypical morphology of the seminiferous tubules in the testes of the HFD fed mice. Additionally, HFD-induced obesity significantly increased the level of aberrant ITO of the seminiferous tubules and significantly lowered the spermatids/spermatocytes ratio. However, the number of Sertoli cells, testicular volume and weight were not affected by 24 weeks of HFD feeding, despite the lower spermatids/spermatocytes ratio. Interestingly, the lowered spermatids/spermatocytes ratio could be compensated by a higher expression of Leydig cells. In more detail, Holm et al. found in human testis biopsies that Leydig cell clusters of more than 15 cells in testicles exhibiting altered testicular structure (Sertoli-cell-only syndrome) or with impaired spermatogenesis^51^. Moreover, they found that the presence of Leydig cell clusters was most pronounced in the testes with an increased luteinizing hormone/testosterone ratio, and that a high proportion of testicular tissue with Leydig cells was associated with a decreased spermatogenic capacity^51^. Leydig cells, responsible for the production of testosterone, express leptin receptors and their function and expression level might be affected by hyperleptinemia^52^. Altogether, HFD probably impaired testicular structure by hyperleptinemia, impaired BTB and membrane fluidity and altered functioning of tight junctions, Sertoli cells and Leydig cells. Future research should examine the expression and function of Leydig cells and tight junctions in relation to HFD-feeding.

In agreement with previous studies, we found that HFD-induced obesity did not affect the total number of the Sertoli cells, spermatocytes and spermatids^53–55^. Additionally, our findings confirmed the importance of the nursing function of Sertoli cells for proper development of sperm cells^8,47,48,56^. Thereby, most probably HFD-feeding did not only affect the quantity of sperm produced, but the also the quality of spermatozoa^22–24^. We found that HFD-induced obesity is associated with a prolongation in stage VII and VIII of spermatogenesis. In more detail, during the stage VII and VIII of seminiferous epithelial cycle of spermatogenesis, important events such as spermatogonial differentiation, initiation of meiosis, initiation of spermatid elongation, and release of fully developed elongated spermatids take place^45,57^. During these critical events the cell adhesion proteins have to reorganise in, for example, the preleptotene stage, where spermatocytes have to enter the BTB and to release the elongated spermatozoa to the lumen of the tubule^58,59^. As mentioned before, increased leptin levels can directly affect testicular structure causing aberrant intra-tubular organisation^43^. Furthermore, high leptin levels can affect the HPG-axis and high free testosterone levels might disrupt the nurturing properties of Sertoli cells^10,44^. Moreover, abnormal testosterone levels can affect the regulatory role of Sertoli cells and specifically the production of adhesion molecules and subsequently the release of spermatozoa in the spermatogenic cycle^60,61^. In addition, lipid content and membrane fluidity modulate maturation of spermatozoa^62^. These described processes could affect spermatogenesis and delay spermatogenesis in this critical stage of the spermatogenic cycle. However, regarding the quality of spermatozoa, indicated by DNA-damage or chromatin condensation, we found no significant difference between dietary groups. Future research should focus on biological processes that can particularly affect the spermatids/spermatocytes ratio and above all the critical processes in stage VII and VII of spermatogenesis, for instance hyperleptinemia, abnormal testosterone levels, lipid composition and membrane fluidity affected by HFD-feeding in the testis. Finally, in future studies the exact biological processes for the spermatogenic delay at the critical events, mostly during spermiation in stages VII and VIII should be enlightened, for instance in adhesion proteins and membrane fluidity and lipid content.

Translational rodent models are the cornerstones for biological studies of human disease pathogenesis. Interestingly, the human disease pathogenesis and key biological pathways and metabolomics are not always totally reflected in commonly used rodent models. For instance, C57BL/6 mice need a very long period of 45% HFD feeding (> 24 weeks) to induce hypercholesteremia and hypertriglyceridemia, which are two key factors in human obesity. Other studies showed that many commonly used rodent models for human pathologies like NAFLD^63^, obesity^64^ or cardiovascular disease^65,66^ did not represent key biological pathways in human pathologies^63^. Therefore, we selected the Ldlr^−/−^.Leiden mouse model of which we certainly know it reflects key pathways and metabolomics in human obesity^67,68^, like adipose tissue inflammation, hypercholesteremia, hyperlipidemia and insulin resistance^33,34,36,68,69^. Notably, the lack of the Ldlr can increase cholesterol levels compared to C57BL/6j mice^35,70^, which might influence normal spermatogenesis^71^. Moreover, many studies that have investigated obesity in relation to male fertility use an ob/ob mouse model. The ob/ob mouse is leptin deficient, whereas leptin is strongly increased in humans with obesity^72^, thus devoid of a factor which affects normal spermatogenesis^17,73–75^. Therefore, future research should in detail examine the role of the Ldlr and leptin deficiency in normal spermatogenesis, particularly tight junctions, Sertoli cells and Leydig cells.

## Conclusion

This study has demonstrated that 24-weeks of HFD-feeding in the Ldlr^−/−^.Leiden mice induced impaired testicular structure, which was associated with an altered spermatids/spermatocytes ratio. Furthermore, HFD-feeding could prolong the spermatogenic cycle in stage VII and VII when four critical events in spermatogenesis take place. Our findings substantiate the emerging evidence of the negative impact of obesity on male fertility and stress the importance of lifestyle guidelines in treatment of male infertility.

## Materials and methods

### Animals and conditions

Male transgenic Ldlr^−/−^.Leiden mice originated from SPF breeding stock at TNO Leiden (TNO Metabolic Health Research, Leiden, The Netherlands) were group-housed in digitally ventilated cages (DVC, Tecniplast S.p.A., Buguggiate, Italy) in conventional animal rooms (relative humidity: 50–60%, temperature: ± 21 °C, light cycle 7 a.m.–7 p.m.) at the central animal laboratory, Radboud university medical center, the Netherlands. The mice had an ad libitum access to food and acidified tap water. The Ldlr^−/−^.Leiden mouse model was chosen because when fed a HFD, adult Ldlr^−/−^. Leiden mice are prone to develop diet-induced obesity, adipose tissue inflammation, hypercholesteremia, hyperlipidemia and insulin resistance^33,34,36^, reflecting key pathways and metabolomics similar to those in human obesity^67,68^.

The experimental approach was approved by an independent institutional ethical committee on animal care and experimentation [Centrale Commissie Dierproeven (CCD), The Hague, The Netherlands], approval number: AVD5010020173464, Radboud university project number: 2017-0063-001). All experiments were performed in accordance with relevant guidelines and regulations. Moreover, statistical power analyses were conducted to minimise the number of animals used in this study, and the ARRIVE guidelines of experimental performance and reporting were met^76^.

At weaning (3 weeks of age), 30 male Ldlr^−/−^.Leiden pups were divided over cages using block-randomisation, resulting in 3–4 animals per cage. Litters were never in the same cage and equally divided over both dietary groups. All mice were fed a standardised reference diet (Sniff R/M-H diet V1530, Sniff Spezialdiäten GmbH, Soest, Germany) until 12 weeks of age. Then, the mice were randomly divided into two dietary groups (control or HFD/intervention group and 15 mice in each group), the control group (Control, n = 15) maintained the standardised reference diet (Sniff, Germany) until the end of the study (36 weeks of age). The HFD group received a 45% lard high fat diet [HFD, D12451, Research diets, New Brunswick, USA (Supplementary data, Table S2)] for 24 weeks (HFD, n = 15), until 36 weeks of age. In this study, individual body weight was measured weekly, and percentage of body fat was indicated by dividing the total fat depots (epididymal, mesenteric and inguinal) weight by body weight at sacrifice.

### Plasma analyses

After 5 h of fasting (8 a.m.–1 p.m.), blood was collected by a tail vein incision, after which EDTA plasma was isolated. Plasma analyses were performed using standardized protocols and assays^34,77,78^. In more detail, total plasma levels of cholesterol and triglycerides were determined using enzymatic assays (CHOD-PAP and GPO-PAP respectively; Roche Diagnostics, Almere, the Netherlands). Total plasma levels for leptin were analysed using standardized protocols and standardized ELISA kits (R&D Systems, Inc., Minneapolis, MN, USA)^34^. Total testosterone concentrations were measured by LC–MS/MS after solid phase extraction^79^.

### Histological analysis of the testis

At the age of 36 weeks, the mice were anaesthetised (3.5% isoflurane, Nicholas Primal (I) limited, London, UK) and sacrificed by transcardial perfusion with 0.1 M phosphate-buffered saline (PBS, 7.3 pH, room temperature). The testes were immediately harvested after euthanasia. The left testis was snap-frozen in liquid nitrogen and preserved at − 80 °C. The right testis was fixed overnight in 4% paraformaldehyde and preserved in 0.1 M PBS with 0.01% sodium azide at 4 °C.

The right testis was weighed (g) and the length, depth and width was measured in mm, and an ellipsoid formula (volume = 0.52 × depth × length × width) was used to calculate testis volume^80,81^. Then the testis was embedded in paraffin in vertical position^80,81^. The embedded testis-tissue was sliced with a microtome into 4 µm thick sections. In the middle of the testes, four paraffin sections were selected with an interval of approximately 60 µm, and stained with haematoxylin and eosin (H&E). These H&E stained sections were used for morphological analysis of the testicular structure^45,82^. Per mouse four testis sections were analyses using a standardized grid and method to randomly select 13 seminiferous tubules per paraffin section using Stereo Investigator setup of Microbrightfield bioscience (MBF Bioscience, Williston, USA). Subsequently, the relative and absolute number of seminiferous tubules in the testis were measured by using the program Fiji (Fiji (Is Just ImageJ), maintained by Eliceiri/LOCI group, University of Wisconsin-Madison, USA & the Jug and Tomancak labs, Dresden, Germany). The thickness of seminiferous epithelium was indicated by measuring the width of the epithelium with Fiji (52 epithelia per mouse). Furthermore, the testicular sections were evaluated for the presence of different types of atypical tubules (Supplementary Fig. S1). For all 52 seminiferous tubules per mouse, the number of tubules in which the germ cells were loosely arranged (hereafter referred to as ‘aberrant intra-tubular organisation’ or AITO), were counted. Testis with a severe level of loosely arranged germ cells [Sertoli-cell-only syndrome (SCO)] were excluded from the analyses (n = 2 Control group and n = 1 HFD group, Supplementary Fig. S1).

The number of Sertoli cells, primary spermatocytes and elongating spermatids were counted in the midsection (four sections of 4 µm thick, interval of 60 µm) of the testis in 52 seminiferous tubules per mouse per mouse (see Supplementary Fig. S2). All quantifications and the nature of the section were analysed using standardized methods and by a blinded expert. For the analysis of the development of the germ cells, the ratio of elongating spermatids over spermatocytes was manually calculated. In these same tubules the stage of spermatogenic development was determined as well, following Oakberg’s description^83^.

### TUNEL and CMA3 staining

To evaluate the consequences of the previous described delay in spermatogenic development at the level of sperm chromatin condensation and DNA damage, we performed a terminal deoxynucleotidyl transferase dUTP nick end labelling (TUNEL) assay^84^ to examine DNA damage and fragmentation in testis sections and evaluated chromatin condensation (CMA3+) in epididymal spermatozoa. In four paraffin testis sections per mouse, the TUNEL assay was performed using the DeadEnd Colorimetric TUNEL System from Promega (Promega Corporation, Madison, USA) following the manufacturer’s protocol^84^. After staining the sections were rinsed in running tap water for 1 min, dehydrated with 99.5% ethanol and xylene and mounted with quick-D mounting medium^84^. Labelled (brown coloured) apoptotic cells were manually counted for all 52 seminiferous tubules per mouse using Stereo Investigator setup of Microbrightfield bioscience (MBF Bioscience, Williston, USA) at 20 × magnification.

To examine sperm chromatin condensation, a chemical staining with chromomycin A3 (CMA3) was performed using a standardised protocol^85^, as this compound binds to DNA if not fully compacted. Spermatozoa were collected from the epididymis of the left-side snap-frozen testis. The obtained sperm sample was then diluted with freezing medium and cryopreserved in liquid nitrogen. For the CMA3 staining, the frozen sperm samples were thawed, fixated with methanol and acetic acid on slides, stained, air-dried, and mounted with VECTAshield (Vector Laboratories, Inc. Burlingame, United States).

The proportion of damaged cells (TUNEL assay) or positive for the CMA3 staining was analysed by counting the number of positive stained cells per field, using brightfield and fluorescence microscopy (I3, excitation BP 450–490 nm, emission LP515) respectively. For the CMA3 staining, an average of 250 spermatozoa (with a minimum of 200 cells) per proband was counted and the percentage of CMA3-positive cells was calculated. Of detail, two samples showed an extreme high percentage of CMA3 + cells, which we could not explain on a biological ground. We therefore we decided to exclude these samples for the CMA3 + analysis. For the TUNEL quantification, the number of positive cells and the developmental stage of the germ cell was registered. Two testis sections per mice were completely scanned for TUNEL-positive cells and the percentage of TUNEL-positive cells in relation to the number of seminiferous tubules present in the sections was calculated.

### Statistical analysis

Throughout the whole study, a random and blocked, double-blind selection procedure was applied. The results are presented as mean ± standard error of the mean (SEM). The means were analysed using one-way Analysis of Variance (ANOVA) in a statistical program, SPSS25 (IBM SPSS Statistics 25, IBM Corporation, Armonk, New York, USA). Correlation tests were performed with Pearson’s correlations. Due to skewed distribution, the testes weight and percentage loosely arranged tubules were log transformed. No equal variances were assumed for the plasma testosterone levels and were therefore statistically analysed in the non-parametric Mann–Whitney *U* test and correlated using the Spearman rho’s test. The results were referred to as significant if the P-value was 0.05 or lower (p- and F-values are both presented in the results).

## Supplementary Information

Supplementary Information.
