# Supplementary material for: High fat diet-induced obesity prolongs critical stages of the spermatogenic cycle in a Ldlr−/−.Leiden mouse model
Source: Sci Rep. 2022 Jan 11;12:430. doi: 10.1038/s41598-021-04069-y (PMC8752771; doi:10.1038/s41598-021-04069-y)
Supplement: Supplementary file 1 — Supplementary Information. [file 41598_2021_4069_MOESM1_ESM.docx]

**Supplementary data**

**Table S1**. Additional mean values of the testis analyses

|  | **Control-group** | **HFD-group** |
| --- | --- | --- |
| Testis weight | 101,45 mg | 100,08 mg |
| Testis volume | 268,31 mm^3^ | 269,85 mm^3^ |
| Relative number of tubuli in testis section | 57,6 % | 57,1 % |
| Thickness of seminiferous epithelium | 5,61 µm | 5,10 µm |

**Table S2. Dietary composition of the high fat diet used in this study.** HFD: high fat diet (D12451, Research diets Inc., New Brunswick, USA). Proportions of compounds are presented within the diet mixture as percentages based on mass (gm%) and kilo calories (kcal %).

|  | HFD | |
| --- | --- | --- |
|  | % (w/w) | kcal % |
| Protein | 24.0 | 20.0 |
| Carbohydrates | 41.0 | 35.0 |
| Crude fat  fat | 24.0 | 45.0 |
| Total |  | 100 |
| kcal/g |  | 4.73 |
|  |  |  |
| Ingredients |  |  |
| Casein | 23.3 | 19.7 |
| L-Cystine | 0.3 | 0.3 |
| Crude fibre | 5.8 | 0 |
| Corn starch | 8.5 | 7.2 |
| Maltodextrin-10 | 11.7 | 9.9 |
| Sucrose | 20.1 | 17.0 |
| Lard | 20.7 | 39.4 |
| Soybean oil | 2.9 | 5.5 |
|  |  |  |
| Minerals & vitamins |  |  |
| Mineral mix S10026 | 1.16 | 0.0 |
| Vitamins mix V10001 | 1.16 | 0.9 |
| Di-calcium phosphate | 1.51 | 0.0 |
| Calcium carbonate | 0.64 | 0.0 |
| Potassium citrate | 1.92 | 0.0 |
| Choline bitartrate | 0.23 | 0.0 |

**
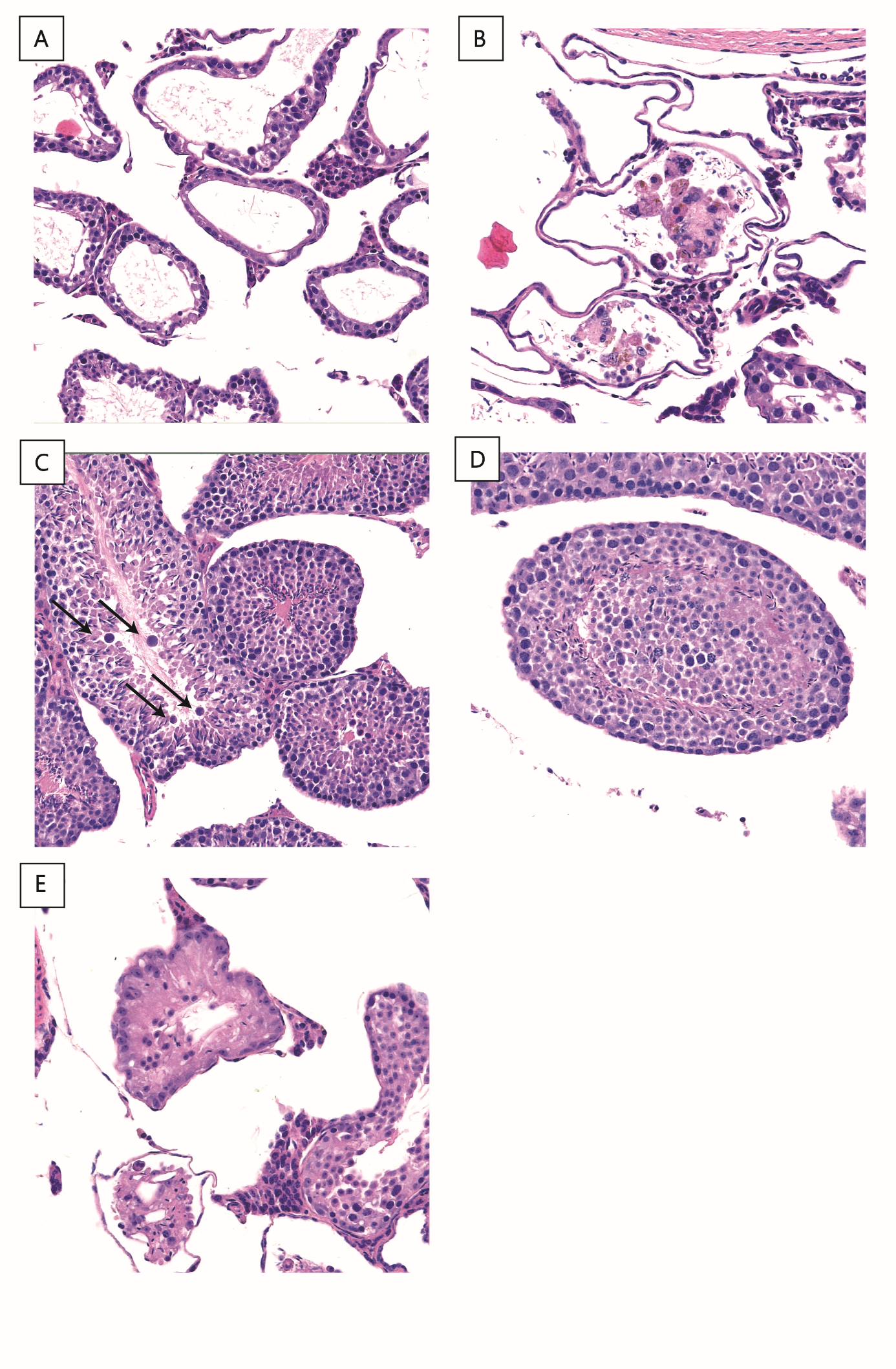
**

**Figure S1. Atypical seminiferous tubules.** (**A**) Tubular dilation. (**B**) Multicellular structures in dilated tubules. (**C**) Atypical cells in lumen of tubules. (**D**) Tubule in stasis. **E**) Atypical/Sertoli cell only (SCO) tubules.


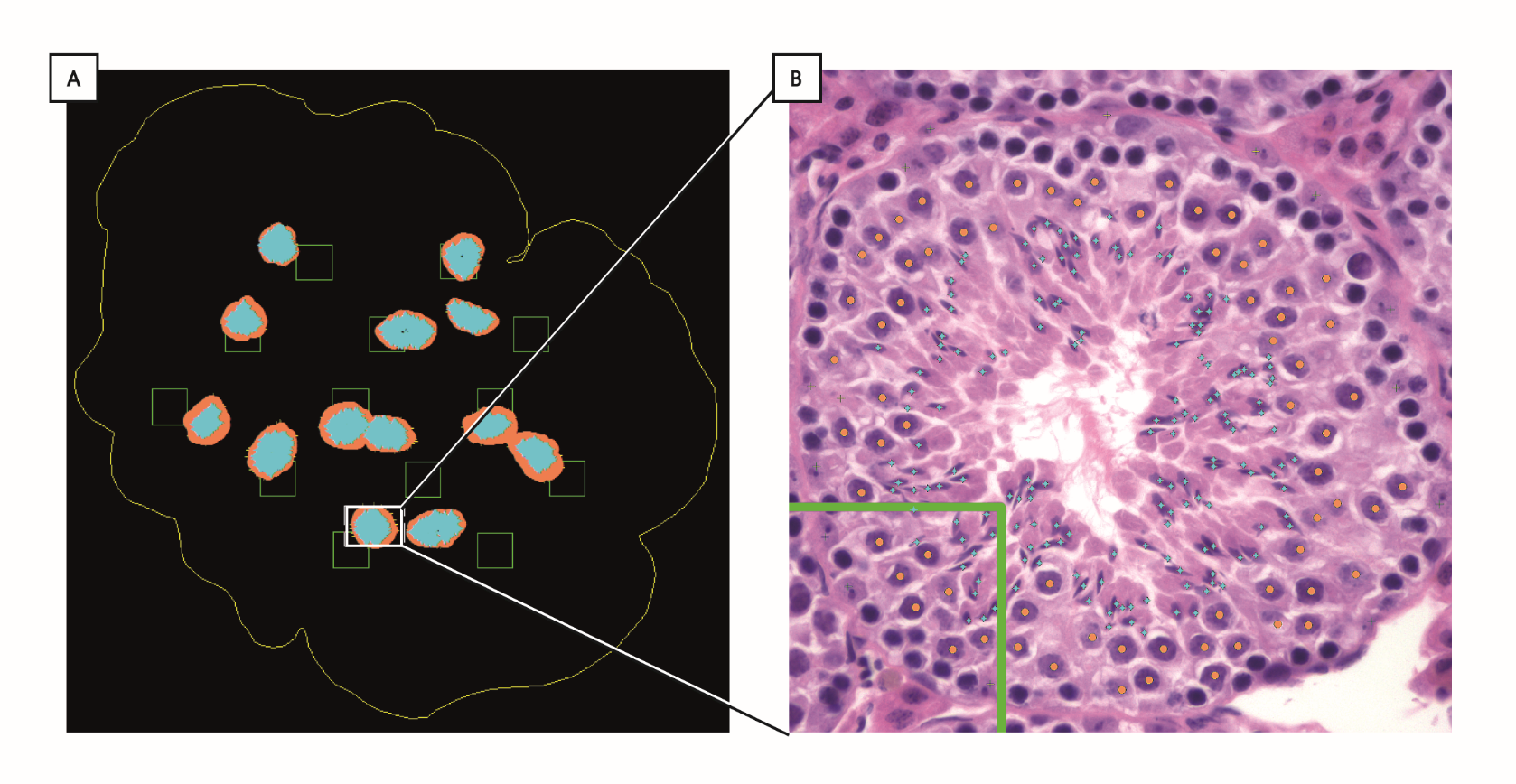


**Figure S2. Quantification of different cell types in testis using the program Stereo investigator.** (**A**) Overview of the counting grid. (**B**) Example counting. Blue diamond shape: elongating spermatids; Red circle: spermatocytes; Yellow plus: Sertoli cells. (Green lines in lower left corner are the grid lines). Magnification x400.


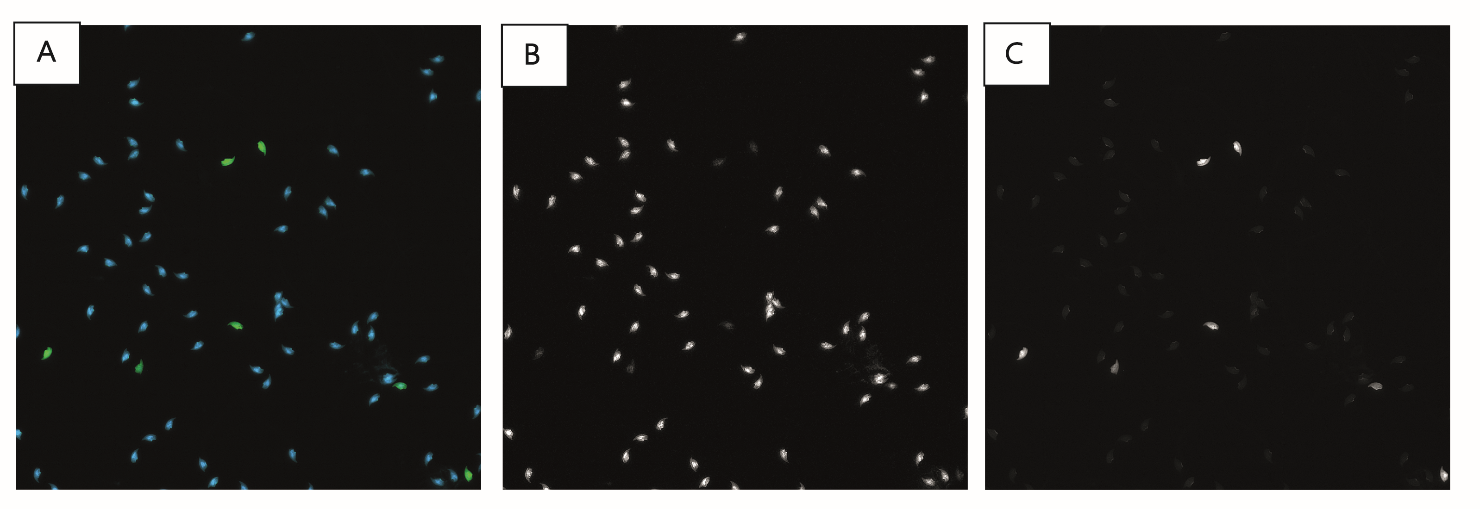


**Figure S3. Split channels for CMA3 stained spermatozoa.** (**A**) Example of a CMA3 staining with the CMA3-positive spermatozoa (green) and the CMA3-negative spermatozoa (blue). (**B**) CMA3-negative spermatozoa. (**C**) CMA3-positive spermatozoa.
